# Supplementary material for: Acoustic Analogues of High-Index Optical Waveguide Devices
Source: Sci Rep. 2018 Jul 10;8:10401. doi: 10.1038/s41598-018-28679-1 (PMC6039535; doi:10.1038/s41598-018-28679-1)
Supplement: Supplementary file 1 — Supplementary figures [file 41598_2018_28679_MOESM1_ESM.pdf]

Supplementary Figures for the paper

Acoustic Analogues of High-Index Optical Waveguide Devices

Farzad Zangeneh-Nejad and Romain Fleury*

Laboratory of Wave Engineering, EPFL, 1015 Lausanne, Switzerland

* *Email: romain.fleury@epfl.ch*

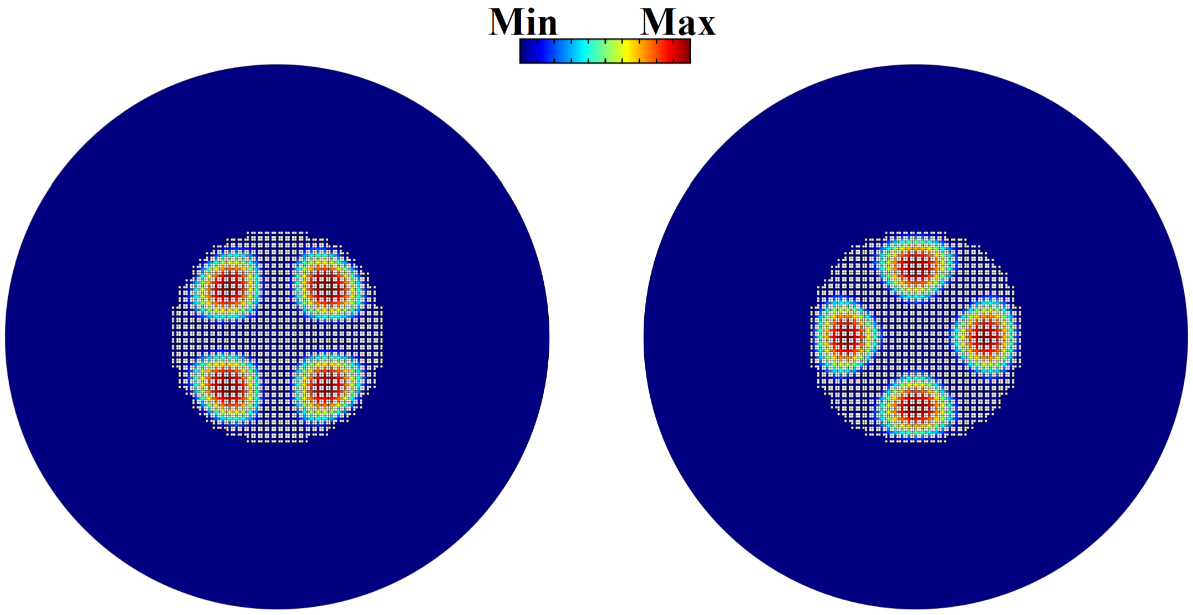

Supplementary Figure 1: Third order guided mode of the acoustic fiber. The field patterns are akin to that of LP_{21} mode in an optical fiber. The two represented profiles are degenerate.

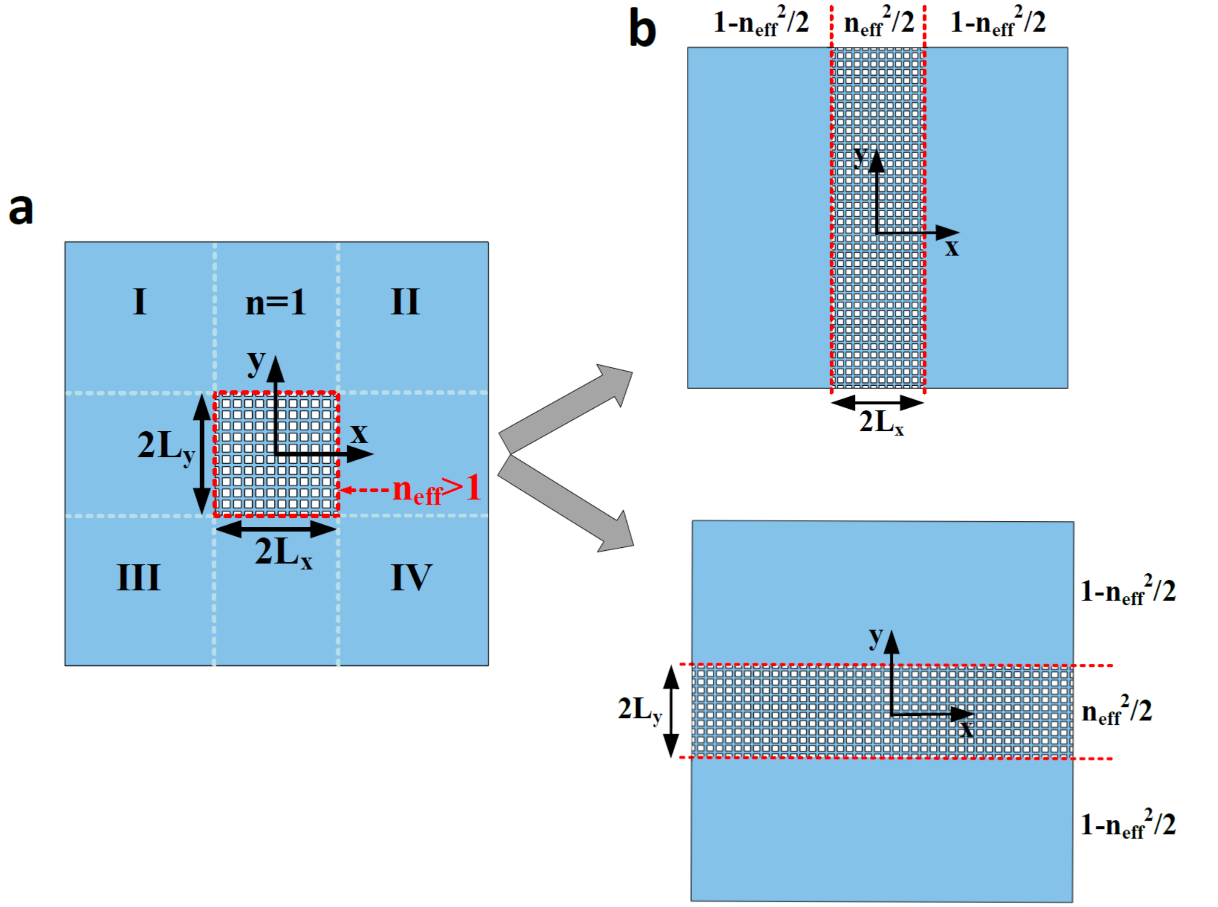

Supplementary Figure 2: Kumar's method for obtaining the characteristic equation of guided modes in the acoustic rectangular waveguide, a, Acoustic rectangular waveguide we aim to analyze: a finite piece of our metamaterial is truncated to a rectangular cross section whose length and width are assumed to be L_x and L_y, and is surrounded by air. The whole geometry is considered to be infinite along z. **b**, Kumar's approach for obtaining the propagation constant of guided modes: the rectangular waveguide is reduced to two independent slab waveguides, one is solely dependent on x (top panel) whereas the other is solely dependent on y (bottom panel). All we need is to calculate β_x and β_y, and then use the relation $\beta^2 = {\beta_x}^2 + {\beta_y}^2$.

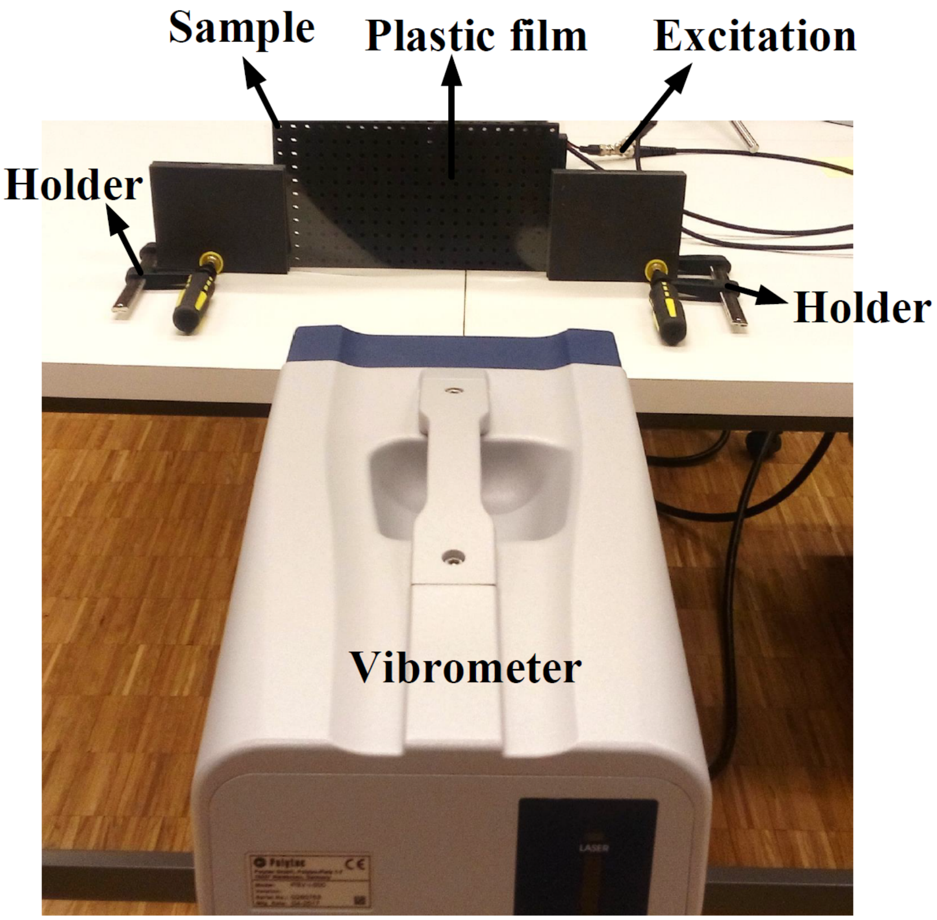

Supplementary Figure 3: Experimental setup used to achieve the field profile of the guided mode of our slab waveguide. The sample has excited from the back side. The resulting change in the pressure induces small fluctuation in the plastic film located in front of the sample. A Polytech PSV-500 vibrometer then measures these fluctuations and scans the full area of the sample to make the field profile of interest.
